# Bacterial and Archaeal Communities Change With Intensity of Vegetation Coverage in Arenized Soils From the Pampa Biome

**DOI:** 10.3389/fmicb.2019.00497

**Published:** 2019-03-22

**Authors:** Camille E. Granada, Luciano Kayser Vargas, Bruno Brito Lisboa, Adriana Giongo, Caroline Thais Martinho, Leandro de M. Pereira, Rafael R. de Oliveira, Fernanda Bruxel, Elisete Maria de Freitas, Luciane M. P. Passaglia

**Affiliations:** ^1^Programa de Pós-graduação em Biotecnologia, Universidade do Vale do Taquari – Univates, Lajeado, Brazil; ^2^Laboratório de Solos, Fundação Estadual de Pesquisa Agropecuária (FEPAGRO), Porto Alegre, Brazil; ^3^Instituto do Petróleo e dos Recursos Naturais. Av. Ipiranga, Pontifícia Universidade Católica do Rio Grande do Sul (PUCRS), Porto Alegre, Brazil; ^4^Pontifícia Universidade Católica do Rio Grande do Sul (PUCRS), Escola de Ciências. Av. Ipiranga, Porto Alegre, Brazil; ^5^Universidade do Vale do Taquari – Univates, Programa de Pós-Graduação em Sistemas Ambientais Sustentáveis, Lajeado, Brazil; ^6^Departamento de Genética, Instituto de Biociências, Universidade Federal do Rio Grande do Sul (UFRGS), Porto Alegre, Brazil

**Keywords:** arenization, dystrophic acid soil, degradation, bacterial community, plant composition, Pampa Biome

## Abstract

Arenization occurs in regions that present sandy soils with normal rainfall levels. Predatory use of environmental sources, the dissolution of arenitic rocks and reworking of non-consolidated surface sands intensify this degradation scenario. Thus, this work aimed to evaluate the impact of the arenization process in the Brazilian Pampa Biome and how this phenomenon affects the soil microbial and plant communities. For this purpose, three arenized areas in Southern Brazil (Pampa Biome) were selected and, in each one, three sampling points were studied: arenized (ARA), arenized to grassland transition (AGT), and grassland (GRA) areas. In the three sampling points, soils presented low levels of nutrients, organic matter, mud and pH acidic in all regions but, the presence of vegetation coverage in AGT and GRA areas preserved the topsoil structure. Our study related ARA with bacterial families *Alcaligenaceae*, *Pseudomonadaceae*, and *Xanthomonadaceae*. AGT with bacterial families *Bacillaceae* and *Burkholderiaceae*, and plant species *Melinis repens* (Willd.) Zizka and *Paspalum stellatum* Humb. and Bonpl. ex Flüggé, and GRA with bacterial families *Koribacteraceae*, *Hyphomicrobiaceae*, and *Chthoniobacteraceae*, and plant species *Croton subpannosus* Müll.Arg. ex Griseb., *Piptochaetium montevidense* (Spreng.) Parodi and *Elyonurus* sp. The three studied areas (as well as sampling points) present soils extremely poor in nutrients with sandy texture, and the bacterial and plant composition well known to be resistant to environmental stresses were dominant. The vulnerability of these areas causes a degradation scenario, which is worsened by agricultural activities. However, in general, this phenomenon is a natural process that occurs mainly due to soil characteristics (poor soils) and climatic variations.

## Introduction

Arenization is a phenomenon related to a range of land degradation processes that mostly impact the productivity, as vegetation degradation, water and wind erosion, salinization, soil compaction, and soil fertility decline (losses of clay, silt, and organic matter), which causes sand deposits without vegetation (Carvalho, 2006; Roesch et al., 2009). This degradation scenario presents a similar pattern than those shown in desertified areas (United Nations Environmental Program [UNEP], 1992), although exhibiting normal rainfall levels (approximately 1,400 mm annual) and sandy soils. The predatory use of environmental sources, the dissolution of arenitic rocks and reworking of non-consolidated surface sands enhance arenization process (Martini, 1979; Roesch et al., 2009). On a local scale, this phenomenon was observed, for example, in Southern Brazil (Overbeck et al., 2007), Northwest Portugal (Sequeira Braga et al., 2002), at the Venezuela’s mountains (Martini, 1979), western and northern Australia (Dregne, 2002), in South Africa (Hoffman and Todd, 2000).

In South Brazil, the Pampa Biome presents soils originated from sedimentary rocks, mainly sandstones, which make them very fragile and liable to the arenization process (Roesch et al., 2009). It is characterized by native grassland, with sparse shrub and tree formations, where the livestock is the main socioeconomic activity (Overbeck et al., 2007). Arenized areas start with degradation scenario characterized by losses of clay, silt and organic matter in small portions of soil, and natural (rainfall and wind) and human activities (agriculture and overgrazing) tend to widen their sizes (Yong-Zhong et al., 2005; Suertegaray and Verdum, 2009), forming sand spots without vegetation surrounded by grassland or native vegetation (Roesch et al., 2009). The transition from arenized to grassland area present reduced vegetation, with significant amounts of exposed soil and plant debris. Although the Pampa Biome presents sandy soils in all the Brazilian extension, there are some differences between arenized area and the grassland which surrounds these sand spots, for example the intensity of vegetation coverage. Besides, it is estimated that more than 5,000 hectares are under the arenization process in Brazilian Pampa Biome areas.

These losses on the vegetation cover entail reduction of plant root exudates and plant decomposing material, causing soil aggregate instability and dissolution (Bronick and Lal, 2005), which results in reductions in microbial biomass (Blankinship et al., 2016; Cagnini et al., 2018) and activity (Granada et al., 2013) by absence of food (Gastine et al., 2003). In these unwelcoming environments, the remaining microbes present specific characteristics that make them highly resistant to adverse conditions while the demand for nutrients decreases (Liu et al., 2016), causing a substantial reduction in nutrient cycling, organic matter turnover and soil aeration (Bissett et al., 2011). As demonstrated by Granada et al. (2013), rhizospheric environments of undisturbed soils, such as those observed in the grasslands from Pampa Biome, keep high microbial biomass and diversity when compared with rhizospheric environments in areas with reduced vegetation cover (arenized). Bacteria with the potential to be classified as Plant Growth Promoting (PGPB), that probably helps plant development and establishment in those inhospitable areas, are among the most affected organisms (Lehman et al., 2015).

It has already been demonstrated that the plant root system may control abiotic parameters such as soil nitrogen concentration, moisture and porosity, which modify soil microbial composition and diversity (Bronick and Lal, 2005). The loss of vegetation observed in arenized areas probably affect all ecosystems subjected to the degradation process. Thus, this work aimed to evaluate the impact of the arenization process in the Pampa Biome portion located in the south of Brazil and how this phenomenon affects the soil characteristics, as well as its microbial and plant communities. For this purpose, three arenized areas were chosen to evaluate physicochemical soil characteristics, composition and diversity of plant and microbial communities.

## Materials and Methods

### Sampling Points

**FIGURE 1:**
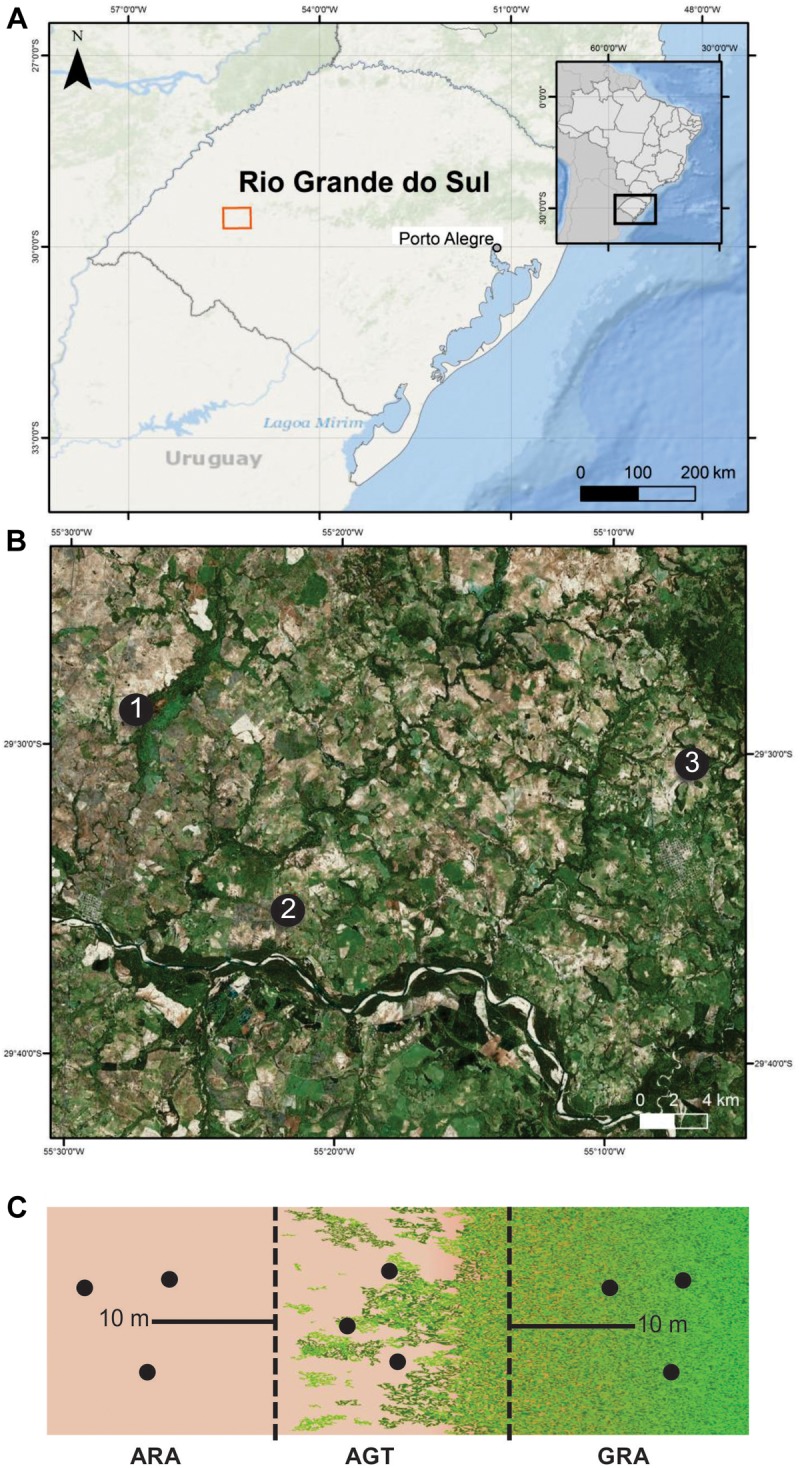
Sampling areas in Pampa Biome, southern Brazil. **(A)** geographic overview; **(B)** areas “1,” “2,” and “3” are indicated by black dots; **(C)** sampling schematic diagram of the arenized (ARA), arenized to grassland transition (AGT) and grassland (GRA) sampling points for each “1,” “2,” and “3” areas. Sampling was carried out in triplicates (black dots). The maps presenting “a” and “b” were constructed in ArcGis software and schematic diagram presented in “c” in Inkscape^®^ software.

Three arenized areas in the south of Brazil were selected (Figure 1A,B). These areas were chosen because they are representative of strategic economic zones, which are suffering an intensive arenization process. In each one of the regions (1, 2, and 3), three sampling points were collected in a gradient profile that ranges from arenized (ARA), arenized to grassland transition (AGT), and grassland (GRA) areas (Figure 1C). The soil samples were collected in March 2015 [total rainfall in the previous month was approximately 123 mm (official data from Secretary of Agriculture and Livestock of the Rio Grande do Sul State)]. They were composed of five to seven randomly selected subsamples, collected with a spade from 0 to 20 cm surface layer (Figure 1C). These soils were packed on ice until reaching the laboratory. The fresh soil was used for microbiological study and dried soil was used to determine the organic matter (OM), sodium (Na), phosphorous (P), potassium (K), zinc (Zn), Copper (Cu), manganese (Mn) contents, and pH using standard methods (Sparks, 1996).

### Grain Size Analysis

Around 50 g of each soil sample were dry sieved in intervals of 1∅. All samples mostly comprised sand; thus, it was not necessary to complete any specific analysis for the fine grain size fraction. The products of sieving were weighted and archived. Mass balance was executed, for each sample, in percentage distribution values. Results of the grain size distribution were converted in statistic parameters values (mean diameter, standard deviation, asymmetry and kurtosis) by the grain size analysis program GRADISTAT v. 8.0 (Blott and Pye, 2001).

### Microbial Biomass and Respiratory Activities

Microbial biomass C (MBC) and respiratory activity (MRA) were determined with 50 g of sieved soil from each sub-sample. The methodology described by Horwath et al. (1996) that uses the soil chloroform fumigation and incubation method was used. The metabolic quotient (qCO_2_) was calculated as the ratio of microbial biomass respiratory to activity.

### Microbial Community Analysis

One gram of fresh soil from each collected sample was subjected to total DNA extraction using the NucleoSpin Soil kit (Macherey-Nagel). Polymerase Chain Reaction (PCR) was used to amplify a partial segment of *16S rRNA* gene (region V4) using the F515 (5′ -GTGCCAGCMGCCGCGGTAA – 3′) and R806 (5′ – GGACTACVSGGGTATCTAAT – 3′) primers (Bates et al., 2011). The amplification reactions contained approximately 5.0 ng of DNA, 0.1 mM MgCl_2_, 20 μM each dNTP, 0.3 μM each primer and 1U of Platinum Taq DNA polymerase (Invitrogen) in a final volume of 25 μL. The amplifications were performed in a PCR Express Temperature Cycling System (Thermo Hybrid) as follows: an initial denaturation step at 95°C for 3 min followed by 25 cycles at 95°C for 30 s, 52°C for 1 min, 72°C for 1 min and one cycle at 72°C for 7 min for final elongation. PCR products were analyzed by electrophoresis in 1% agarose gels in TBE buffer with ethidium bromide and visualized by UV light. The ∼250 base pairs (bp) *16S rRNA* gene fragments obtained were subjected to high-throughput sequencing in an Ion PGM System (Thermo Fisher) following the manufacturer’s protocol.

The *16S rRNA* reads were submitted to quality control that retained sequences with a minimum length of 100 bp and trimmed to remove low-quality bases for the minimum Phred score of 30 and to remove any sequence with ambiguous bases and homopolymers (PRINSEQ; Schmieder and Edwards, 2011). Prior the global clustering, the remaining sequences were dereplicated, sorted by decreasing read abundance and then filtered to exclude singletons (734,564 singletons, corresponding to 51.2% of the sequences at this step) using USEARCH v7.0.1090 (Edgar, 2013). After, 222 putative chimera sequences were removed (which corresponds to 3.4% of the sequences at this step) using the RDP reference database (Cole et al., 2014) in USEARCH. The taxonomic assignment was obtained using QIIME v1.7 (Caporaso et al., 2010). Operational taxonomic units (OTUs) were selected based on a 97% sequence similarity, and taxonomic data through the classification algorithm using the 97% OTUs version of GreenGenes 13.8 (DeSantis et al., 2006). The original sequences were deposited at GenBank under the accession number BioProject PRJNA400135.

### Plants Community Structure

The plant community structure survey in the studied areas (ARA, AGT, and GRA) involved the distribution of 25 sample units of 0.25 m^2^ along transects, maintaining a distance of one meter between each sample unit and each transect.

In each sample unit the surface occupied by the horizontal projection of the aerial part of each species, the exposed soil and the plant dead material were estimated using the surface method (Poissonet and Poissonet, 1969). Each plant species in the sample unit was classified; the absolute and relative coverage, frequency parameters and the importance value index (IVI) were calculated. Sampling sufficiency was estimated by bootstrap in Estimate S software, v. 9.1.0 from a presence-absence matrix (Colwell, 2006).

### Statistical Analysis

A Two-way ANOSIM test was used to verify significant differences among the matrices generated in each one of the three sampling points (ARA, AGT, and GRA) analyzed, and significant differences were followed by the SIMPER test. The eight bacterial families and the eight plant species that most contributed to variability (calculated by SIMPER test), and five environmental parameters [pH, Mud, organic matter (OM), exposed soil and plant dead material] were chosen for a Canonical Correspondence Analysis (CCA) performed with the Past3 software (Hammer et al., 2001). Alpha diversity was re-calculated using rarefaction with maximum depth based on the lower number of representative sequences among the samples (1,415). Shannon diversity index (*H’*) was estimated based on the cover values of the plant species and prokaryotic OTUs in each sampling point.

Cluster analysis (UPGMA) was performed using all soil physicochemical parameters, bacterial population and plant composition both in the Past3 software (Hammer et al., 2001).

One-Way ANOVA, with means compared through Tukey’s test (*p* < 0.01) using the software Assistat 7.6 beta (Silva and Azevedo, 2016) was used to compare the soil physicochemical properties, microbial respiratory activity (MRA), microbial biomass (MBC), metabolic quotient (qCO_2_), exposed soil (ES) and Shannon diversity indexes in ARA, AGT and GRA areas. Non-parametric tests Kruskal–Wallis and Mann–Whitney tests (*p* < 0.01) were used to compare bacterial genera.

## Results

The studied areas presented poor soils with a sandy texture, where livestock is the main socioeconomic activity. The grain size analysis classified the sediment in average as unimodal moderately to moderately well sorted fine sand. Soils from ARA and AGT regions were unimodal moderately well sorted fine sand with very low content of fine-grained sediments (mud < 62 μm) (Table 1). The physicochemical characteristics of soils from the ARA, AGT, and GRA areas showed a low percentage of mud and organic matter (Table 1), confirming their sandy texture. The overall soil pH was acidic (∼4.5) and the nutrient level was low, mainly in ARA samples. The presence of grassland in the studied areas did not change the physicochemical characteristics of the soils (Table 1). Microbial respiratory activity (MRA) increased from ARA to AGT to GRA and percentage of exposed soil (ES) decreased in the same order (Table 2). Parameters MRA and ES were linearly correlated (*R* = -0.8737, *p* ≤ 0.001). Metabolic quotient (qCO_2_) was lower in ARA (0.8) and similar in AGT and GRA (2.2 and 2.0, respectively) soils.

**Table 1:** Physicochemical characteristics in the soil samples from Arenized (ARA), Arenized to Grassland Transition (AGT), and Grassland (GRA) areas.

Sampling point	Mud	Na	OM	pH	Zn	Cu	Mn	P	Al	K^∗^
			
	——- % ——-				————– mg/dm^3^ ————-					
ARA	0.3 ± 0.4	1.0 ± 0	0.9 ± 0.4	4.6 ± 0.3	0.2 ± 0.2	0.3 ± 0.1	3.4 ± 2.2	2.8 ± 2.4	0.5 ± 0.3	6.3 ± 0.6b
AGT	0.8 ± 0.1	1.0 ± 0	1.3 ± 0.4	4.5 ± 0.3	0.4 ± 0.1	0.3 ± 0.1	5.5 ± 2.9	9.3 ± 6.4	0.5 ± 0.2	18.7 ± 9.3ab
GRA	2.4 ± 2.1	1.7 ± 0.6	1.2 ± 0.2	4.4 ± 0.2	0.4 ± 0.1	0.5 ± 0.1	8.4 ± 2.6	1.8 ± 1.2	0.5 ± 0.1	30.7 ± 4.7a


*Each observed value is the media of three areas studied ± standard deviation. ^∗^Significant statistical differences was showed by different letters (only in K content) according to ANOVA, with means compared via the via Tukey’s test (p < 0.01). OM, Organic Matter; Zn, Zinc; Cu, Copper; Mn, Manganese; P, Phosphorus; Al, Aluminum; K, Potassium.*

**Table 2:** Microbial Biomass (MBC), Microbial Respiratory Activity (MRA), Metabolic quotient (qCO_2_) and Exposed soil (ES) from Arenized (ARA), Arenized to Grassland Transition (AGT) and Grassland (GRA) areas.

	MBC	MRA	qCO_2_	ES
			
	———– C mg (soil kg)^-1^ ———–			——% ——
ARA	82.5 ± 45.7bc	47.0 ± 25.4c	0.8 ± 0.8b	76.3 ± 8.7a
AGT	75.3 ± 16.1b	163.6 ± 31.1b	2.2 ± 0.3a	33.1 ± 2.7b
GRA	178.8 ± 48.1a	269.63 ± 66.3a	2.0 ± 0.2ab	9.7c ± 6.1c


*Statistical analysis was performed by ANOVA, with the means followed by different letters differ by using Tukey’s test (*p* < 0.01 for MRA and ES; 0.01 ≤*p* ≤ 0.05 for MBC and pCO_2_).*

**FIGURE 2:**
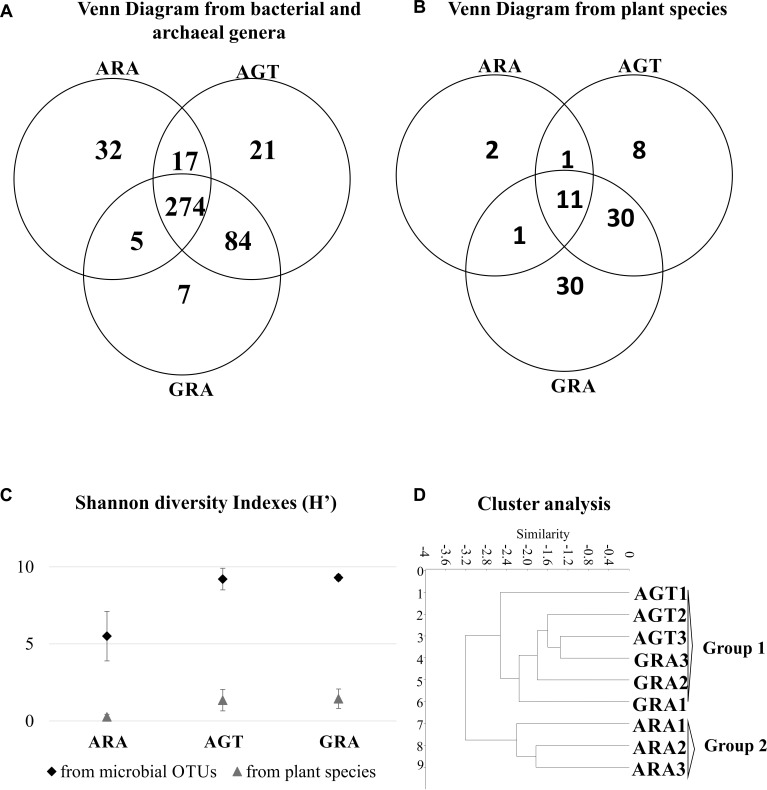
Similarities between ARA, AGT and GRA sampling points. **(A)** Venn diagram showing absolute numbers of unique and shared microbial operational taxonomic units (OTUs) between ARA, AGT and GRA sampling points; **(B)** Venn diagram showing numbers of unique and shared plant species between ARA, AGT and GRA sampling points; **(C)** Shannon diversity index of microbial genera and plant species; **(D)** similarities using UPGMA distances.

The arenization process affects directly the diversity of plants and microorganisms. Microbiological analysis identified a total average of 4195 OTUs (average of 1,277 for ARA; 5,482 for AGT and 5532 for GRA – Supplementary Table S1). A total of 83 different plant species and 440 different bacterial genera were found in the three analyzed areas. In the ARA area, were found 14 plant species and 328 different bacterial genera; AGT had 50 plant species and 396 bacterial genera and GRA counted 72 plant species and 370 bacterial genera (Figure 2A,B). The Shannon diversity index for prokaryotic OTUs and plant species (Figure 2C) was similar in GRA and AGT (9.3 and 9.2 for bacteria and 1.4 and 1.3 for plants, respectively) and lower in ARA (5.5 for bacteria and 0.3 for plants), possibly indicating that the microbial diversity changed with the presence/absence of vegetation coverage. Cluster analysis relating soil physicochemical parameters, plant and bacterial composition (Figure 2D) grouped the regions according to the sampling points (GRA, AGT, and ARA). The sampling points with cover plants (GRA and AGT) presented similar characteristics and formed one group. The region without cover plants (ARA) grouped separately.

**FIGURE 3:**
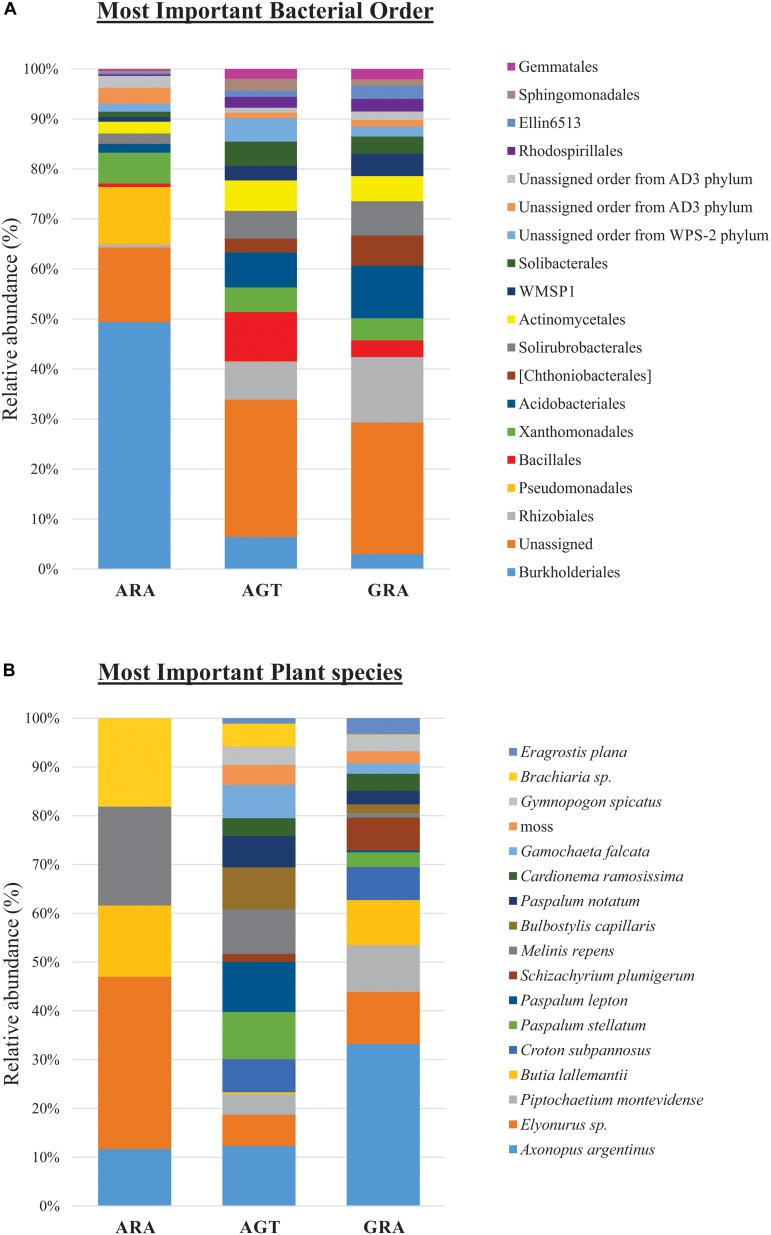
Relative abundance of microbial orders and plant species. **(A)** Percentage of operational taxonomic units (OTUs) sorted in the 19 principal microbial orders and **(B)** 17 principal plant species.

Bacterial and archaeal community were distributed within 31 phyla, 84 classes, 160 orders, 263 families (or candidate divisions), and 440 genera. The candidate divisions are new microbial taxa not yet described in the literature, but whose sequences were found during metagenomics or metabarcoding surveys. In the three analyzed areas, were identified 156 bacterial orders (or candidate divisions) and four archaeal orders (Nitrososphaerales, Cenarchaeales, Methanobacteriales and a candidate division). The archaeal order Cenarchaeales was found only in ARA and AGT samples, and Methanobacteriales only in ARA samples. The 19 bacterial orders that contributed with more than one percent for total diversity (calculated by SIMPER test) are shown in Figure 3A. Six out of 19 bacterial orders were candidate divisions or unassigned. The orders Burkholderiales and Rhizobiales were the most important for total diversity (responsible for 28.4 and 7.3% of the total, respectively). The relative abundance of microbial orders belonging to the Rhizobiales, Acidobacteriales, Chthoniobacterales, Solirubrobacterales, candidate division WMSP1 and Rhodospirillales decreased toward to degraded area (GRA to AGT to ARA). On the other hand, the relative abundance of the orders Burkholderiales, Pseudomonadales, and Xanthomonadales increased toward degraded area. In ARA samples, Burkholderiales (47.3%) and Pseudomonadales (11%) were predominant, accounting for almost 60% of total identified OTUs.

Among the 83 plant species identified, only five were non-native of Pampa Biome [*Melinis repens* (Willd.) Zizka, *Brachiaria* sp., *Eragrostis plana* Nees, *Cardionema ramosissima* (Weinm.) A. Nelson and J. F. Macbr. and *Cerastium commersonianum* Ser.]. The factors that most contributed to cover diversity were percentage of exposed soil (28.8%), presence of *Axonopus argentinus* Parodi (10.3%), dead plant material (7.0%) and presence of *Elyonorus* sp. (4.4%). The 17 plant species that contributed with more than one percent for total diversity (SIMPER test) are shown in Figure 3B (their relative abundance in each sampling point are listed in the Supplementary Table S2). In ARA samples, only five different plant species were found (*Axonopus argentinus*, *Elyonurus* sp., *Butia lallemantii* Deble and Marchiori, *Melinis repens* and *Brachiaria* sp.), and all 17 plants were found in AGT and GRA samples. *Axonopus argentinus* and *Elyonurus* sp. were the two most abundant plant species in GRA (33.2 and 10.7%, respectively), *Axonopus argentinus* and *Paspalum lepton* Schult. were the two most abundant plant species in AGT (12.3 and 10.3%, respectively), and *Elyonurus* sp. and *Melinis repens* were the two most abundant plant species in ARA (35.3 and 20.2%, respectively).

**FIGURE 4:**
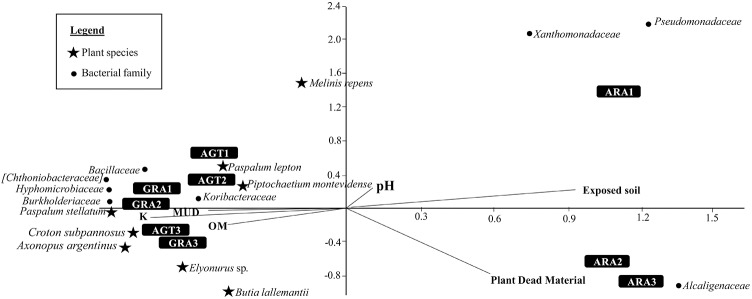
Canonical Correspondence Analysis for the eight bacterial families and eight plant species that most contributed to variability (calculated by SIMPER test), and five environmental parameters [pH, Mud, organic matter (OM), exposed soil and plant dead material].

The Canonical Correspondence Analysis is shown in the Figure 4. Degraded areas observed in ARA are related to big areas of exposed soil and high amounts of plant dead material. These areas are also related with bacterial families *Alcaligenaceae*, *Pseudomonadaceae*, and *Xanthomonadaceae*. AGT and GRA are related with a little more amount of Organic Matter (OM), K, and Mud. Both areas are related with bacterial families *Bacillaceae, Hyphomicrobiaceae, Koribacteraceae, Chthoniobacteraceae*, and *Burkholderiaceae*, and plant species *Paspalum stellatum* Humb. and Bonpl. ex Flüggé, *Croton subpannosus* Müll. Arg. ex Griseb., *Piptochaetium montevidense* (Spreng.) Parodi and *Elyonurus* sp. (relative abundances of principal microbial families in each sampling point are listed in the Supplementary Table S3).

The high-throughput sequencing showed that the number of unclassified OTUs (not yet described in literature) increased in the areas with highest bacterial diversity (AGT and GRA). Among the 10 bacterial genera that most contributed for the total diversity, five were unclassified at genus level (Table 3). Bacterial genera *Pseudomonas*, *Stenotrophomonas* and the unclassified OTUs from the family *Alcaligenaceae* were found in relative abundance higher than 0.1% only in ARA, and unclassified OTUs from *Chthoniobacteraceae* and *Sinobacteraceae* families were found only in AGT and GRA samples. Regarding rare microbiota (relative abundance ranging from 0.1 to 0.01% of the total OTUs in at least one sample), 133 genera were observed in samples. Half of these genera are known in literature as potential plant growth promotion bacteria (PGPB), which can present at least one plant growth promotion trait, such as nitrogen fixation, phosphate solubilization, manganese oxidation, siderophore production or auxin biosynthesis. *Balneimonas, Beijerinckia*, *Bacillus*, *Ferruginibacter*, *Methylobacterium*, *Variovorax*, and *Sphingomonas* are examples of genera with plant growth promotion traits found in low abundance.

**Table 3:** Ten bacterial genera that most influenced the total soil microbial diversity (calculated by SIMPER test) in the three analyzed areas.

Phylum; Class; Order; Family; Genus (or respective operational taxonomic unit, *OTU*)	Contribution (%)	ARA	AGT	GRA
Proteobacteria; Betaproteobacteria; Burkholderiales; *Alcaligenaceae*; Unclassified OTU	8.0	14.3 ± 8.2a	0.1 ± 0b	0 ± 0b
Proteobacteria; Gammaproteobacteria; Pseudomonadales; *Pseudomonadaceae*; *Pseudomonas*	6.7	10.4 ± 1.9a	0 ± 0b	0 ± 0b
Proteobacteria; Alphaproteobacteria; Rhizobiales; *Hyphomicrobiaceae*; *Rhodoplanes*	4.4	0.3 ± 0.1b	2.8 ± 0.9a	6.6 ± 3.3a
Acidobacteria; Acidobacteriia; Acidobacteriales; *Koribacteraceae*; Unclassified OTU	3.4	1.1 ± 0.8c	2.9 ± 0.9b	5.9 ± 0.7a
Alphaproteobacteria; Gammaprotebacteria; Xanthomonadales; *Xanthomonadaceae*; *Stenotrophomonas*	3.3	5.1 ± 1.1a	0 ± 0b	0 ± 0b
Firmicutes; Bacilli; Bacillales; *Bacillaceae*; *Bacillus*	2.7	0.1 ± 0.1b	3.9 ± 3.5a	1.2 ± 0.8a
Verrucomicrobia; Spartobacteria; [Chthoniobacterales]; [*Chthoniobacteraceae*]; DA101 OTU	2.7	0 ± 0c	1.3 ± 1.1b	4.0 ± 1.7a
Actinobacteria; Thermoleophilia; Solirubrobacterales; *Conexibacteraceae*; Unclassified OTU	2.4	1.5 ± 1.4b	3.6 ± 1.5a	4.1 ± 1.2a
Proteobacteria; Gammaproteobacteria; Xanthomonadales; *Sinobacteraceae*; Unclassified OTU	2.3	0 ± 0b	2.5 ± 0.9a	3.2 ± 0.7a
Proteobacteria; Betaproteobacteria; Burkholderiales; *Burkholderiaceae*; *Burkholderia*	2.3	0.9 ± 1.4	3.4 ± 0.5	1.2 ± 0.6


*Abundances and standard deviation less than 0.05 were rounded to 0. Means followed by different letters differ by Kruskal–Wallis and Mann–Whitney tests (*p* < 0.01).*

## Discussion

The Pampa Biome covers an area in the southern of South America that is spread in Brazil, Argentina and Uruguay (Lupatini et al., 2013; Vargas et al., 2015). This biome is considered priority for flora and fauna conservation due to its huge biodiversity (Overbeck et al., 2013). The presence of intense vegetation coverage (GRA) induces to the idea that the soils of Pampa’s biome are very fertile; however, this work showed that the topsoil structure of studied areas presented low levels of nutrients, organic matter, mud and acidic (Table 1). The extreme sandy texture fosters the natural degradation by the action of water and wind erosion (Weber et al., 2007; Roesch et al., 2009), which was observed in arenized areas (ARA). Vegetation coverage has a fundamental role in maintaining soil moisture. Thus, the absence of vegetation observed in ARA and AGT results in loss of soil moisture, which potentiate wind erosion (Gong Li et al., 2000). GRA soils presented higher average percentage of fine sediments compared to ARA and AGT soils, probably due to its higher amount of vegetation that fixes and prevents fine fraction from being transported by wind. The ARA soil in Area 3 presented a higher average percentage of medium sand content. This may indicate that in this location wind transports finer fractions (<62 μm) and fine sand, and leaves behind coarse-grained sediments as lag deposits. Thus, at Area 3 either the wind is stronger or the arenization process is more developed.

The presence of grassland improves protection for the soil, decreasing erosion and preserving the topsoil structure (Cao et al., 2017). Decrease of plant cover (GRA to AGT to ARA – Table 2) diminished soil MBC and MRA. As already demonstrated, one of the most important factors for microbial growth is the soil carbon availability (Zak et al., 2003) and the presence of plant material in degradation processes and dense rhizospheric environment improved the organic carbon content of the soil (Tu et al., 2006) and consequently, MRA and biomass are also improved (Table 2; Cao et al., 2017). The absence of plant root biomass (also root exudates) in degraded arenized soils (ARA) decreased microbial activity, probably due to starvation and lack of nutrients (Gastine et al., 2003). The sandy soils themselves are not able to keep soil moisture.

Microbial biomass was higher in environments with dense vegetation coverage (GRA) when compared to those with moderate vegetation coverage (AGT), but it did not change in environments with moderate vegetation coverage when compared to those without vegetation coverage (AGT to ARA – Table 2). MRA (MRA – Table 2) is correlated with soil moisture (Singh and Gupta, 1977) and the decrease in MRA observed from GRA to ARA could be explained by reduction/absence of vegetation coverage that reduces water retention in the soils. Metabolic quotient showed that microbial population of AGT and GRA soils was more efficient for executing their metabolic functions than in ARA soils (Granada et al., 2013). The absence of the plant rhizospheric system probably affected the survival of many sensitive bacterial/archaeal genera, reducing the total diversity of microorganisms (Figure 2; Wang et al., 2007; Panda, 2011). Undisturbed soils for long periods (GRA and AGT) presented highly diverse microbial communities (Griffiths et al., 2004), although it is not resistant to soil perturbation (Wittebolle et al., 2009) and as observed in ARA, a few bacteria families dominate total bacterial population (the four most abundant families account for approximately 81% of total OTUs).

Some studies reported that specific soil characteristics are essential to improve microbial diversity. The increase of Al_3_, clay and P in acidic soils were already related to a decrease in microbial diversity (Faoro et al., 2010). Andrew et al. (2012) showed that pH and carbon percentage in desertic soil are related to microbial β-diversity. However, the results presented in this work showed that soils from the three sampling points (ARA, AGT, and GRA) are extremely poor, and no statistical differences among soil characteristics were identified. Because of that, we consider that the decrease of microbial diversity presented from GRA to ARA may be related to the presence of vegetation coverage (rhizospheric environments). The high number of different plant species which comprise native grasslands from Pampa Biome are important in the composition of soil organic carbon (root exudates). These root exudates attract soil microorganisms and enables microbiotic development, which favors the improvement of microbial diversity (Philippot et al., 2013; Mellado-Vázquez et al., 2016; Fierer, 2017).

The environmental degradation observed in ARA soils very likely selected for bacterial families that are well known to present high resistance to adverse conditions, such as *Alcaligenaceae* (Abbas et al., 2015; Fierer, 2017), *Pseudomonadaceae* (Chang et al., 2008), and *Xanthomonadaceae* (Sharan et al., 2008), all of those belonging to Proteobacteria phyla (Figure 4). Some bacterial isolates from Proteobacteria phyla are known as Plant Growth Promoting Bacteria (PGPB) and the predominance of strains belonging to this phylum in desertic soils was already discovered by Jorquera et al. (2012). The most abundant microbial orders in ARA soils, Burkholderiales and Pseudomonadales, account for around 60% of the total identified OTUs. Granada et al. (2013) studied microbial diversity in arenized soils and identified *Burkholderia* as the dominant genus by culture-dependent methods. Fierer (2017) reported that *Burkholderia* is low tolerant to soil stress (limited carbon and nutrients, low pH and others) and highly tolerant to soil disturbance (drying-rewetting, fire and aggregate instability). It might suggest that this bacterial genus can grow rapidly and exploit unoccupied niches generated as a result of biotic or abiotic disturbances (Fierer, 2017), as observed in arenized soils from Pampa Biome.

In our study, less than 20% of identified plants had the ability to grow in arenized regions (ARA; Figure 3). Plants that are subjected to prolonged abiotic stresses, such as water limitation and nutrient starvation, need to developed specific physiological and molecular stress responses allowing them to thrive under normally unfavorable conditions. However, root colonization by microorganisms and their services are also essential for the plant establishment in extreme environments (Soussi et al., 2016; Cao et al., 2017).

As observed by Feng et al. (2015) in desertified areas from China, the combination of rural socioeconomic factors and climatic factors had an important effect on vegetation cover. According to McKinney (2001) and Zenni and Ziller (2011), human population size and occupation rates are positively related to the number of non-native plant species. Thus, non-native plant diversity is closely approximated by a simple model of human population growth and diffusion, since plant introductions occur from landscaping, farming and others (McKinney, 2001). The studied region of Pampa’s biome presented a low percentage of non-native plant species (less than five percent – Freitas et al., 2010) and low human occupation registered in this region (Brazilian Institute of Geography and Statistics [IBGE], 2010) indicated that the occurrence of arenized and degraded areas could be worsened by livestock (as overgrazing), which is the principal activity in the studied region. However, this phenomenon is a natural process that starts mainly due to soil characteristics and climatic variations (rain, wind, and others – Roesch et al., 2009).

In order to recover these arenized areas, the development of restoration/conservation programs which simulate natural processes with a restoration of soil fertility and facilitate vegetation development is necessary (Gao et al., 2011; Cao et al., 2014). To achieve success, this type of program needs high investment and actions for human awareness. Better land use, agricultural management and ecological practices could be helpful to interrupt or slow down the spreading of degraded areas in terrestrial ecosystems. As highlighted by Cao et al. (2014), these types of programs may take some time to become effective, since vegetation establishment needs a long time. So, land managers must be patient to see environmental restoration.

## Data Availability

The datasets generated for this study can be found in NCBI, PRJNA400135.

## Author Contributions

CG, LV, BL, and LMPP conceived the ideas. CG, BL, and EF collected the data. AG, CM, LMP, RO, CG, BL, and FB analyzed the data. CG, LV, AG, and LMPP led the writing.

## Conflict of Interest Statement

The authors declare that the research was conducted in the absence of any commercial or financial relationships that could be construed as a potential conflict of interest.
