# Supplementary material for: Bacterial and Archaeal Communities Change With Intensity of Vegetation Coverage in Arenized Soils From the Pampa Biome
Source: Front Microbiol. 2019 Mar 22;10:497. doi: 10.3389/fmicb.2019.00497 (PMC6439421; doi:10.3389/fmicb.2019.00497)
Supplement: Supplementary file 1 [file Table_1.docx]

Table S1: Overview of the number of sequences and operational taxonomic units (OTUs) in soil from different arenization sites.

|  | **ARA** | | | | | | | | |
| --- | --- | --- | --- | --- | --- | --- | --- | --- | --- |
| **Sample** | **1.1** | **1.2** | **1.3** | **2.1** | **2.2** | **2.3** | **3.1** | **3.2** | **3.3** |
| Input sequences | 2,224 | 146,211 | 114,766 | 62,553 | 36,352 | 33,883 | 95,774 | 5,026 | 126,322 |
| Input mean length | 238.6 | 233.5 | 230.9 | 241.35 | 241.54 | 241.1 | 234.44 | 237.25 | 234.57 |
| Good sequences* | 2,037 | 128,791 | 100,414 | 57,170 | 33,436 | 31,002 | 86,461 | 4,567 | 113,159 |
| Good mean length | 242.43 | 247.85 | 244.8 | 248.31 | 246.07 | 247.19 | 240.07 | 243.98 | 242.86 |
| Representative sequences** | 1,415 | 90,969 | 71,584 | 38,548 | 22,649 | 21,623 | 62,393 | 3,374 | 85,309 |
| Representative OTUs** | 324 | 2,661 | 2,523 | 2,276 | 1,884 | 1,744 | 1,693 | 237 | 811 |
|  | **AGT** | | | | | | | | |
| **Sample** | **1.1** | **1.2** | **1.3** | **2.1** | **2.2** | **2.3** | **3.1** | **3.2** | **3.3** |
| Input sequences | 101,583 | 146,523 | 65,762 | 122,386 | 24,201 | 96,185 | 126,845 | 117,168 | 151,195 |
| Input mean length | 227.48 | 226.41 | 226.74 | 234.09 | 238.9 | 235.98 | 226.96 | 231.99 | 225.27 |
| Good sequences | 87,930 | 125,123 | 57,302 | 109,796 | 22,256 | 87,250 | 109,708 | 104,096 | 129,214 |
| Good mean length | 243.16 | 245.66 | 240.26 | 243.02 | 243.74 | 242.91 | 242.37 | 242.21 | 243.45 |
| Representative sequences** | 50,478 | 78,710 | 31,617 | 65,569 | 13,107 | 51,763 | 61,100 | 61,879 | 77,064 |
| Representative OTUs | 4,845 | 4,327 | 4,177 | 6,658 | 3,521 | 6,227 | 6,184 | 6,513 | 6,889 |
|  | **GRA** | | | | | | | | |
| **Sample** | **1.1** | **1.2** | **1.3** | **2.1** | **2.2** | **2.3** | **3.1** | **3.2** | **3.3** |
| Input sequences | 92,879 | 85,624 | 68,554 | 125,468 | 57,991 | 186,019 | 124,548 | 156,341 | 176,910 |
| Input mean length | 228.57 | 226.95 | 230.67 | 226.68 | 230.8 | 228.45 | 224.2 | 224.29 | 237.63 |
| Good sequences | 80,778 | 74,466 | 60,810 | 108,674 | 51,572 | 161,736 | 107,251 | 133,530 | 159,834 |
| Good mean length | 244.05 | 241.33 | 241.49 | 242.36 | 241.52 | 243.99 | 239.98 | 242.12 | 247.2 |
| Representative sequences** | 48,236 | 42,604 | 35,510 | 62,800 | 29,589 | 96,555 | 66,277 | 81,292 | 106,446 |
| Representative OTUs | 5,549 | 5,563 | 5,084 | 5,912 | 4,394 | 6,297 | 5,347 | 5,596 | 6,048 |

In sample identification the first number is the sampling area, and second number is the replicate. ARA = Arenized area, AGT = transition area, GRA = Grassland; *sequences with a minimum length of 100 bp and minimum Phred score of 30; **representative sequences after USEARCH.
