# Supplementary material for: Bacterial and Archaeal Communities Change With Intensity of Vegetation Coverage in Arenized Soils From the Pampa Biome
Source: Front Microbiol. 2019 Mar 22;10:497. doi: 10.3389/fmicb.2019.00497 (PMC6439421; doi:10.3389/fmicb.2019.00497)
Supplement: Supplementary file 2 [file Table_2.DOCX]

**Table S2:** Relative abundance of 21 plant species which more contribute for total diversity according to the SIMPER test.

|  | **Contribution %** | **ARA** | | |  | **AGT** | | |  | **GRA** | | |
| --- | --- | --- | --- | --- | --- | --- | --- | --- | --- | --- | --- | --- |
| **Taxon** |  | **1** | **2** | **3** |  | **1** | **2** | **3** |  | **1** | **2** | **3** |
| Exposed soil | 28.8 | 86.3 | 72 | 70.5 |  | 32.7 | 30.6 | 35.9 |  | 10.5 | 3.3 | 15.3 |
| *Axonopus argentinus* | 10.3 | 0 | 0 | 1.1 |  | 0.4 | 0 | 13.8 |  | 0.5 | 35.7 | 20.5 |
| Plant dry matter | 7.0 | 4.9 | 21.9 | 20.9 |  | 13.2 | 13.5 | 15.1 |  | 9.5 | 20.8 | 28.2 |
| *Elyonurus* sp. | 4.4 | 0 | 0 | 3.3 |  | 0 | 0 | 7.4 |  | 0 | 2.5 | 15.8 |
| *Butia lallemantii* | 3.2 | 0 | 0 | 1.4 |  | 0 | 0 | 0.5 |  | 0.2 | 0 | 15.7 |
| *Piptochaetium montevidense* | 3.1 | 0 | 0 | 0 |  | 1.5 | 3.3 | 0 |  | 11.4 | 4.9 | 0 |
| *Croton subpannosus* | 2.7 | 0 | 0 | 0 |  | 2.4 | 5.4 | 0 |  | 2.0 | 9.5 | 0 |
| *Paspalum stellatum* | 2.7 | 0 | 0 | 0 |  | 0 | 10.4 | 0.9 |  | 0 | 5.1 | 0 |
| *Melinis repens* | 2.2 | 1.9 | 0 | 0 |  | 9.3 | 1.3 | 0 |  | 1.7 | 0 | 0 |
| *Schizachyrium plumigerum* | 2.1 | 0 | 0 | 0 |  | 0 | 0.3 | 1.6 |  | 0 | 11.4 | 0 |
| *Paspalum lepton* | 2.1 | 0 | 0 | 0 |  | 4.5 | 7.4 | 0 |  | 0.7 | 0 | 0 |
| *Paspalum notatum* | 1.7 | 0 | 0 | 0 |  | 4.8 | 2.7 | 0 |  | 3.9 | 0.8 | 0 |
| *Cardionema ramosissima* | 1.6 | 0 | 0 | 0 |  | 2.6 | 1.2 | 0.3 |  | 5.9 | 0 | 0 |
| *Bulbostylis capillaris* | 1.6 | 0 | 0 | 0 |  | 1.5 | 4.9 | 3.6 |  | 1.6 | 0.9 | 0.5 |
| Moss | 1.4 | 0 | 0 | 0 |  | 0 | 0 | 4.8 |  | 2.7 | 0 | 1.6 |
| *Brachiaria* sp. | 1.3 | 1.7 | 0 | 0 |  | 5.5 | 0 | 0 |  | 0.3 | 0 | 0 |
| *Gymnopogon spicatus* | 1.2 | 0 | 0 | 0 |  | 1.8 | 2.6 | 0 |  | 2.3 | 3.4 | 0 |
| *Eragrostis plana* | 1.2 | 0 | 0 | 0 |  | 1.3 | 0 | 0 |  | 5.7 | 0 | 0 |
| *Gamochaeta falcata* | 1.2 | 0 | 0 | 0 |  | 2.3 | 2.7 | 2.9 |  | 2.1 | 0.3 | 1.2 |
| *Senecio cisplatinus* | 1.0 | 0 | 0 | 0 |  | 3.9 | 0 | 0 |  | 1.9 | 0 | 0 |
| *Solidago chilensis* | 0.9 | 0 | 0 | 0 |  | 0 | 0 | 0 |  | 4.9 | 0 | 0 |
| *Aristida circinalis* | 0.9 | 0 | 0 | 1.6 |  | 0 | 2.8 | 1.3 |  | 0 | 1.1 | 0 |
| *Borreria brachystemonoides* | 0.9 | 0 | 0 | 0 |  | 0 | 0 | 1.8 |  | 0 | 3.5 | 0 |

1 = sampling area 1; 2 = sampling area 2; 3 = sampling area 3.
