# Supplementary material for: Bacterial and Archaeal Communities Change With Intensity of Vegetation Coverage in Arenized Soils From the Pampa Biome
Source: Front Microbiol. 2019 Mar 22;10:497. doi: 10.3389/fmicb.2019.00497 (PMC6439421; doi:10.3389/fmicb.2019.00497)
Supplement: Supplementary file 3 [file Table_3.DOCX]

**Table S3:** Average of abundance of 22 microbial families which contribute with more than 1% for total diversity according to the SIMPER test.

|  | **Contribution %** | **ARA** | | |  | **AGT** | | |  | **GRA** | | | |
| --- | --- | --- | --- | --- | --- | --- | --- | --- | --- | --- | --- | --- | --- |
| **Taxon** |  | **1** | **2** | **3** |  | **1** | **2** | **3** |  | **1** | **2** | **3** |  |
| *Alcaligenaceae* | 25.1 | 20.5 | 30 | 86.8 |  | 0.1 | 0.7 | 0.003 |  | 0.6 | 0.03 | 0 |  |
| *Unassigned* | 8.1 | 10.3 | 26.9 | 5.6 |  | 20.9 | 27.2 | 23.4 |  | 24.2 | 18.6 | 25.3 |  |
| *Pseudomonadaceae* | 6.9 | 32.7 | 0.01 | 0.02 |  | 0 | 0 | 0 |  | 0 | 0 | 0 |  |
| *Xanthomonadaceae* | 4.3 | 17.4 | 0.1 | 0.05 |  | 3.1 | 1.2 | 0.8 |  | 0.9 | 0.6 | 0.05 |  |
| *Hyphomicrobiaceae* | 3.7 | 0.6 | 0.4 | 0.02 |  | 2.9 | 2.2 | 3.8 |  | 2.4 | 9.1 | 9.3 |  |
| *Bacillaceae* | 3.4 | 0.02 | 0.4 | 0.1 |  | 9.8 | 0.3 | 6.0 |  | 0.4 | 2.8 | 1.6 |  |
| *Koribacteraceae* | 3.1 | 2.5 | 2.1 | 0.2 |  | 4.1 | 5.6 | 3.5 |  | 9.1 | 6.0 | 6.6 |  |
| *Chthoniobacteraceae* | 2.7 | 0.04 | 0.1 | 0.02 |  | 1.9 | 3.2 | 1.9 |  | 3.6 | 7.5 | 4.4 |  |
| *Burkholderiaceae* | 2.3 | 2.7 | 0.05 | 0.2 |  | 7.5 | 2.0 | 1.9 |  | 2.1 | 1.3 | 0.6 |  |
| *Conexibacteraceae* | 2.2 | 0.7 | 3.5 | 0.7 |  | 2.4 | 5.5 | 4.3 |  | 5.8 | 3.1 | 5.0 |  |
| *Bradyrhizobiaceae* | 2.1 | 0.1 | 0.1 | 0.1 |  | 3.9 | 1.6 | 3.5 |  | 1.4 | 4.9 | 4.2 |  |
| Unclassified bacteria from phylum AD3 | 1.9 | 1.5 | 7.5 | 0.3 |  | 0.1 | 1.1 | 1.5 |  | 1.8 | 0.5 | 1.3 |  |
| Unclassified bacteria from phylum WPS2 | 1.9 | 1.3 | 2.7 | 0.5 |  | 3.3 | 6.3 | 2.6 |  | 2.5 | 0.9 | 1.7 |  |
| *Sinobacteraceae* | 1.8 | 0.1 | 0.1 | 0.06 |  | 3.3 | 2.1 | 2.5 |  | 2.6 | 3.9 | 3.4 |  |
| Unclassified bacteria from phylum Chloroflexi | 1.7 | 0.2 | 0.7 | 0.1 |  | 0.6 | 3.4 | 1.8 |  | 3.1 | 1.7 | 4.4 |  |
| *Solibacteraceae* | 1.6 | 0.4 | 1.5 | 0.4 |  | 4.6 | 2.5 | 3.8 |  | 2.3 | 2.2 | 2.6 |  |
| Unclassified bacteria from phylum AD3 | 1.4 | 1.7 | 5.1 | 0.01 |  | 0.7 | 1.5 | 0.4 |  | 1.1 | 1.7 | 1.5 |  |
| Unclassified bacteria from phylum Acidobacteria | 1.2 | 0.7 | 0.2 | 0.01 |  | 0.9 | 1.6 | 0.6 |  | 2.9 | 2.8 | 1.0 |  |
| *Acidobacteriaceae* | 1.1 | 0.06 | 0.05 | 0.02 |  | 2.2 | 1.2 | 1.5 |  | 2.4 | 1.2 | 1.8 |  |
| *Rhodospirillaceae* | 1.1 | 0.1 | 0.1 | 0.03 |  | 1.7 | 1.0 | 1.1 |  | 1.2 | 2.8 | 1.8 |  |
| *Planococcaceae* | 1.0 | 0.01 | 0.2 | 0.01 |  | 2.0 | 0.1 | 2.8 |  | 0.2 | 1.1 | 0.7 |  |
| *Streptomycetaceae* | 1.0 | 0.1 | 3.6 | 0.03 |  | 0.6 | 1.1 | 0.6 |  | 0.9 | 0.8 | 0.5 |  |

1 = sampling area 1; 2 = sampling area 2; 3 = sampling area 3.
